# Potential Applications of Remote Limb Ischemic Conditioning for Chronic Cerebral Circulation Insufficiency

**DOI:** 10.3389/fneur.2019.00467

**Published:** 2019-05-03

**Authors:** Jiulin You, Liangshu Feng, Liyang Bao, Meiying Xin, Di Ma, Jiachun Feng

**Affiliations:** Department of Neurology and Neuroscience Center, The First Hospital of Jilin University, Changchun, China

**Keywords:** remote limb ischemic conditioning (RLIC), chronic cerebral circulation insufficiency (CCCI), chronic cerebral hypoperfusion (CCH), pathophysiology, cerebral blood flow (CBF)

## Abstract

Chronic cerebral circulation insufficiency (CCCI) refers to a chronic decrease in cerebral blood perfusion, which may lead to cognitive impairment, psychiatric disorders such as depression, and acute ischemic stroke. Remote limb ischemic conditioning (RLIC), in which the limbs are subjected to a series of transient ischemic attacks, can activate multiple endogenous protective mechanisms to attenuate fatal ischemic injury to distant organs due to acute ischemia, such as ischemic stroke. Recent studies have also reported that RLIC can alleviate dysfunction in distant organs caused by chronic, non-fatal reductions in blood supply (e.g., CCCI). Indeed, research has indicated that RLIC may exert neuroprotective effects against CCCI through a variety of potential mechanisms, including attenuated glutamate excitotoxicity, improved endothelial function, increased cerebral blood flow, regulation of autophagy and immune responses, suppression of apoptosis, the production of protective humoral factors, and attenuated accumulation of amyloid-β. Verification of these findings is necessary to improve prognosis and reduce the incidence of acute ischemic stroke/cognitive impairment in patients with CCCI.

## Overview of Chronic Cerebral Circulation Insufficiency (CCCI)

Chronic cerebral circulation insufficiency (CCCI) refers to a group of clinical symptoms of chronic brain dysfunction that occur due to pathological decreases in cerebral blood flow. As the causes of CCCI are diverse, its precise definition remains controversial. However, researchers agree that chronic cerebral hypoperfusion is the initiating factor (1–3), leading to pathophysiological changes including impaired glucose metabolism and protein synthesis, glutamate excitotoxicity in the central nervous system (CNS), inflammation, autophagy disorders, neuronal apoptosis, and endothelial dysfunction. Long-term chronic cerebral hypoperfusion may eventually lead to impairments in cognitive function (4, 5), affective disorders such as depression (6, 7), and acute ischemic stroke (8, 9).

Despite the lack of consensus, CCCI is often classified according to etiology in clinical practice. In some cases, CCCI occurs due to large vessel stenosis, atherosclerotic stenosis, or occlusion of the intracranial and extracranial large arteries (3, 10–12). Another cause of CCCI is cerebral small vessel disease [e.g., hypertension-induced small vessel disease, vascular amyloidosis, cerebral autosomal dominant arteriopathy with subcortical infarcts and leukoencephalopathy (CADASIL), etc.], insufficient hypoperfusion is more likely to occur in deep white matter structures (4, 13), leading to relatively characteristic changes such as white matter lesions and multiple lacunar infarction. In other cases, CCCI may be caused by circulatory disorders, cardiac or hemodynamic abnormalities such as hypotension, or cardiac insufficiency (14, 15).

Unlike acute ischemic stroke, CCCI exhibits a chronic degenerative course characterized by a series of pathophysiological changes, without acute ischemic necrosis (12). Many patients fail to notice the condition due to an absence of new acute symptoms. However, without treatment or intervention, CCCI may lead to adverse events such as cognitive impairment, depression, and acute ischemic stroke. Therefore, early identification and effective interventions for CCCI are critical for improving patient quality of life and reducing the societal burden. Pharmacological studies have been disappointing. In addition to the treatment for risk factors of CCCI, there is still a lack of effective treatment and drugs (16). Though there are drugs which were found to be protective in preclinical models of CCCI, in reality there has been a major translational block with few agents showing promise in human (13). Thus, more recent studies have begun to focus on stimulating the body's endogenous protection mechanisms via methods such as remote limb ischemic conditioning (RLIC).

## Overview of Remote Limb Ischemic Conditioning (RLIC)

During ischemic conditioning (IC), transient ischemia/reperfusion is artificially induced before or after a lethal ischemic attack to stimulate endogenous protective mechanisms and prevent injury in the ischemic organs or tissues. IC is categorized as either IC *in situ* or RLIC, based on the site of treatment. As early as 1986, Murry et al. reported the protective effect of IC *in situ* against damage associated with acute myocardial infarction. Prior to occlusion of the circumflex coronary artery for 40 min, blood flow was artificially blocked for 5 min, followed by 5 min of reperfusion. After four ischemia/reperfusion cycles, the authors reported significant decreases in the volume of the infarcted myocardium (17). More recent studies regarding acute cerebral ischemia have revealed that IC *in situ* can effectively reduce neural cell death and infarct size; attenuate cerebral edema; improve cerebral circulation; and relieve inflammation, apoptosis, and oxidative stress (18). Despite these findings, IC *in situ* is invasive, and there are safety risks associated with subjecting the tissue to additional ischemia. Furthermore, IC *in situ* is difficult in patients who are unsuitable for or reject angioplasty or stenting surgery. Therefore, researchers have begun to focus on RLIC, particularly in the bilateral upper limbs, which exhibit a greater tolerance to ischemia. Previous studies (19, 20) have demonstrated that RLIC can significantly reduce infarct volume and improve neurological function in experimental models of stroke (19, 20). Clinical trials have also yielded promising results (8, 9, 21, 22). One recent study reported that four cycles of intermittent limb ischemia (i.e., 5 min of inflation to 20 mmHg above systolic blood pressure alternated with 5 min of deflation using a standard upper arm blood pressure cuff) is well tolerated and improves National Institutes of Health Stroke Scale (NIHSS) scores in patients with acute cerebral ischemia (21). Moreover, chronic limb hypoperfusion caused by pre-existing arterial peripheral vascular disease (PVD) is capable of inducing neuroprotective effects against acute ischemic stroke in patients who have not undergone surgical treatment (23). In addition to its protective effects in the heart and brain, RLIC has been shown to exert protective effects in several other organs such as the lungs, kidneys, and liver (24). While RLIC applications are currently limited to cases of acute ischemia, recent studies have revealed the protective effects of RLIC against the effects of chronic decreases in blood supply in the brain (8, 9, 25–27) and heart (28), such as those associated with CCCI and stable coronary artery disease.

**Figure 1:**
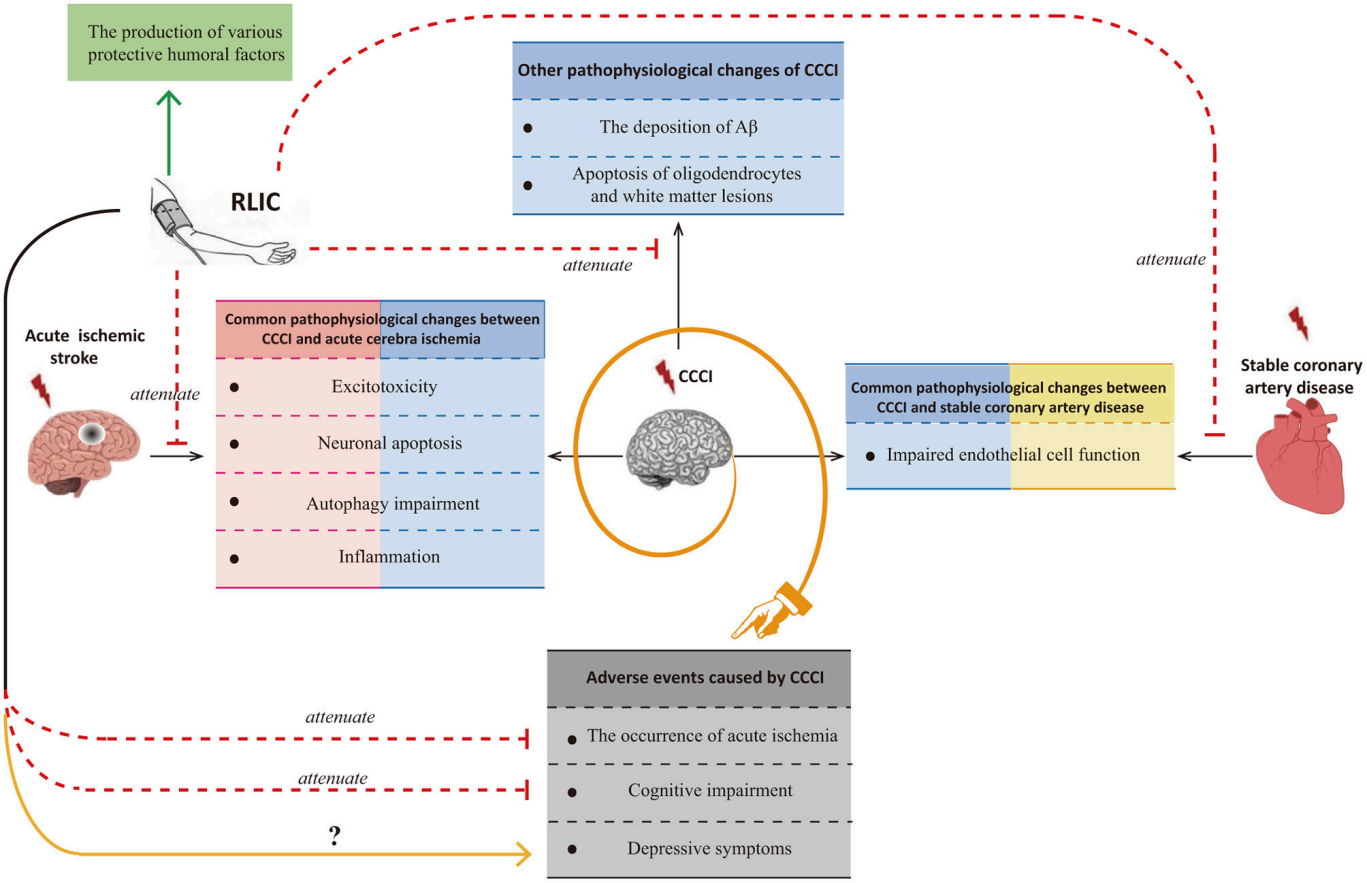
Summary of the effects of RLIC on the brain.

Although researchers have observed that RLIC exerts neuroprotective effects against CCCI, few studies have been performed, and the mechanisms underlying its effects remain poorly understood. Many of the pathophysiological processes associated with CCCI are also observed in patients with acute ischemic stroke or stable coronary artery disease. Glutamate excitotoxicity, neuronal apoptosis, autophagy impairment, and inflammation are known to occur in both CCCI and acute cerebral ischemia, while endothelial dysfunction is known to occur in both CCCI and stable coronary artery disease. Thus, the mechanisms by which RLIC protects against acute ischemic stroke and stable coronary artery disease may be similar to those for CCCI. However, CCCI is also associated with unique pathophysiological changes, including deposition of amyloid-β, apoptosis of oligodendrocytes, and white matter lesions. In addition to inhibiting both the shared and unique pathophysiological changes associated with CCCI, RLIC may also induce the production of a variety of protective humoral factors. Based on the above mechanisms, RLIC can improve the outcomes of CCCI. Although some studies have indicated that RLIC can reduce the incidence of acute cerebral ischemia and improve cognitive function, few studies have investigated whether RLIC can influence the incidence of depression in patients with CCCI (Figure 1).

## RLIC May Regulate Various Pathophysiological Processes Associated With CCCI Caused by Intracranial and Extracranial Stenosis

Pathophysiological changes associated with CCCI caused by large vessel stenosis include glutamate excitotoxicity, neuronal apoptosis, autophagy dysfunction, inflammation, and endothelial cell dysfunction, which are also observed during the course of acute ischemic stroke or chronic myocardial ischemia.

### From Acute Ischemic Stroke to CCCI Caused by Large Artery Stenosis

Previous studies have indicated that RLIC may attenuate a variety of pathophysiological processes and exert neuroprotective effects against acute cerebral ischemia. Thus, RLIC may exert similar effects in CCCI.

#### RLIC Can Alleviate Glutamate Excitotoxicity in the CNS

Glutamate is an important excitatory neurotransmitter in the CNS. Under normal physiological conditions, there is a dynamic balance between the production and clearance of glutamate, ensuring that extracellular glutamate concentrations remain low. Excessive extracellular glutamate accumulation is observed under various pathological conditions (e.g., ischemic stroke, CCCI, and some chronic neurodegenerative diseases) due to increased release and reduce clearance (29–34). Excessive glutamate can stimulate NMDA receptors, AMPA receptors, and some other glutamate receptors on neurons and glial cells, leading to the collapse of electrochemical gradients, the activation of protein kinases and endonucleases, and the degradation of important substances. Such excitotoxicity results in neuronal death, mediates damage to oligodendrocytes, and causes pathological changes such as leukoaraiosis (35).

In the 1980s, researchers revealed that rats subjected to acute focal cerebral ischemia exhibit excessive accumulation of extracellular glutamate in the striatum (36). Subsequent studies reported that excitatory amino acid antagonists (e.g., NMDAR antagonists) can exert neuroprotective effects in such animal models (37). Notably, CCCI due to large artery stenosis is also associated with glutamate excitotoxicity. Vicente et al. (38) evaluated cognitive function and hippocampal slices after 10 weeks in a rat model of permanent common carotid artery occlusion (2VO). The authors reported that 2VO resulted in significant cognitive dysfunction, as well as significant reductions in the rate of glutamate uptake in hippocampal slices. Moreover, a positive correlation was observed between impairments in cognition and neurotoxicity due to excessive extracellular glutamate.

A recent study demonstrated that RLIC can upregulate the expression of glutamine synthetase (GS) during ischemic stroke, promote the utilization of glutamate, and attenuate extracellular glutamate accumulation (39). These findings suggest that RLIC may exert neuroprotective effects in CCCI by alleviating glutamate excitotoxicity.

#### RLIC Can Suppress Apoptosis

Neuronal apoptosis is commonly observed in variety of CNS diseases, including acute cerebral ischemia (39, 40) and CCCI due to large artery stenosis, representing an important cause of cognitive dysfunction (26, 41). While the Bax gene is an important pro-apoptotic gene, Bcl-2 plays a role in the inhibition of apoptosis. The Bcl-2/Bax ratio is commonly used to assess the apoptotic state. Cheng et al. observed a large number of apoptotic cells in the ischemic penumbra of rats subjected to middle cerebral artery occlusion (MCAO), noting that such changes were accompanied increases in levels of Bax protein and decreases in levels of Bcl-2 protein (40). Stanojlović et al. observed a similar phenomenon in a 2VO rat model, which mimics CCCI due to large artery stenosis (41). These findings suggest that inhibition of apoptosis can exert neuroprotective effects during both acute cerebral ischemia and CCCI (26, 40).

Previous research has indicated that RLIC can inhibit neuronal apoptosis in the acute cerebral ischemic area by upregulating Bcl-2 protein levels and downregulating Bax protein levels, thereby increasing the Bcl-2/Bax ratio (40). The mechanisms underlying these effects may involve the activation of multiple signaling pathways. Cheng et al. (40) reported that RLIC promotes the phosphorylation of STAT3, suggesting that changes in the Bcl-2/Bax ratio and decreases in apoptosis are due to transduction of the JAK2-STAT3 signaling pathway. However, another study reported that RLIC inhibits the phosphorylation of STAT3 in the mouse brain, thereby promoting recovery of neurological function following ischemic stroke (39). Thus, the role of the JAK2-STAT3 pathway in the CNS remains controversial, and the discrepant results suggest that it plays different roles in different types of cells. In astrocytes and microglia, transduction of the JAK2-STAT3 pathway may induce reactive astrocytic activation (39), microglia-mediated cell apoptosis, and neurological deficits (42). In neurons, however, the JAK2-STAT3 pathway exerts protective effects against apoptosis (43). Further studies are required to determine whether RLIC exerts protective effects in the ischemic brain via activation of the JAK2-STAT3 pathway.

Additional studies have revealed that RLIC activates the PI3K-pAkt pathway to induce cardioprotective effects, significantly upregulating the Bcl-2/Bax ratio by increasing p-Akt levels and the p-Akt/Akt ratio (44). Similar findings have been observed in models of MCAO (45), indicating that this process may be uniform across different diseases. CCCI due to large artery stenosis leads to decreases in p-Akt levels and the Bcl-2/Bax ratio in the cytoplasm and synapses of hippocampal neurons, suggesting that inhibition of Akt phosphorylation decreases the Bcl-2/Bax ratio and levels of neuronal apoptosis. RLIC may promote the phosphorylation of p-Akt and upregulate the Bcl-2/Bax ratio by activating the PI3K-pAkt pathway, thereby inhibiting neuronal apoptosis during CCCI.

#### RLIC Can Regulate Inflammatory Responses, Including the Inflammation of Local Brain Tissue, and Systemic Immune Status

**Figure 2:**
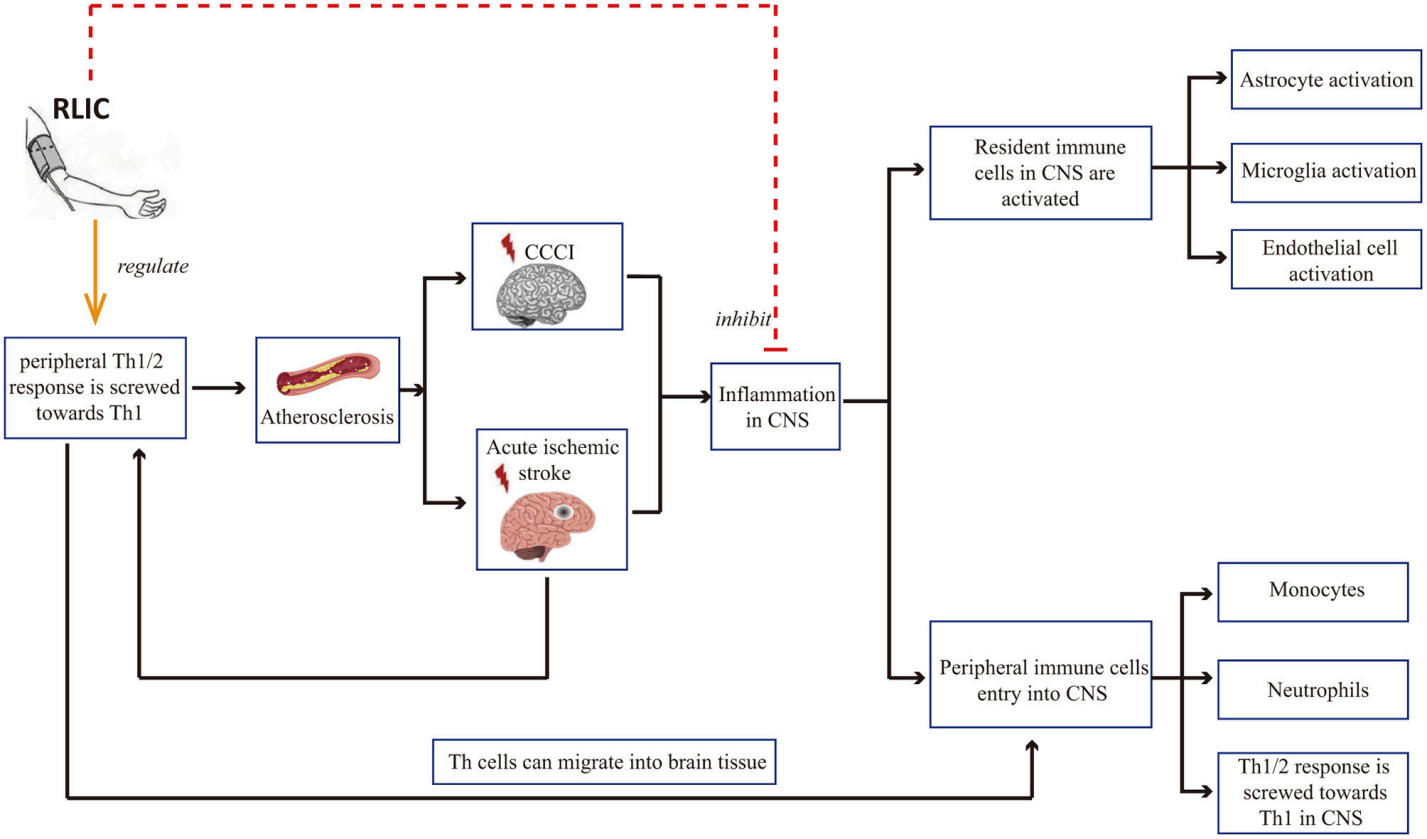
RLIC can regulate inflammatory responses. Th1 cells in the blood contribute to the development of cerebral artery atherosclerosis, which can lead to chronic cerebral hypoperfusion and acute cerebral ischemia, further leading to inflammation of the CNS, including the activation of resident immune cells, the infiltration of peripheral immune cells into the CNS, and the shift of Th1/Th2 balance to Th1 response. RLIC exerts an immunomodulatory effect. In periphery, the immunomodulatory effect of RLIC is controversial. In the CNS, RLIC inhibits the activation of resident immune cells and the infiltration of peripheral immune cells into the CNS, and inhibits the shift of Th1/Th2 to Th1 response.

Inflammation is a common pathophysiological reaction in several CNS diseases. Both the activation of resident immune cells in the CNS and the infiltration of peripheral immune cells contribute to inflammation in the CNS (46–50). Therefore, reducing the severity of the inflammatory response in the brain is critical for neuroprotection. In addition, there is a correlation between peripheral immune status and damage to brain tissue: Acute cerebral ischemia is known to affect the peripheral immune state, leading to a shift in the balance between T helper 1 (Th1)/Th2 cells toward Th1 polarization. Th1 cells in the circulating blood may in turn enter the CNS, thereby exerting pro-inflammatory effects. In addition, Th1 cells in the blood contribute to the progression of atherosclerosis, which is among the causes of CCCI and stroke. Research has indicated that RLIC can regulate inflammatory responses, including local inflammation of the brain tissue and systemic immune status (Figure 2).

##### RLIC can inhibit the activation of resident immune cells in the CNS

Microglia and astrocytes are important resident immune cells in the CNS. Both can be rapidly activated by acute cerebral ischemia (48), releasing multiple inflammatory mediators such as tumor necrosis factor α (TNF-α) and interleukin-1β (IL-1β) (46). Microglial and astrocyte activation has also been observed in the brains in animal models of CCCI (50), as indicated by increased expression of CD11b and Iba1 in microglia as well as increases in the number of astrocytes. In addition, 2VO models exhibit increases in the expression of ICAM-1 and VCAM-1, which are markers of the vascular inflammatory response (51).

Cheng et al. (39) recently reported that RLIC significantly inhibits ipsilateral increases in the expression of glial fibrillary acidic protein (GFAP) following acute ischemia, suggestive of attenuated astrocyte activation. In rats with CCCI due to bilateral common carotid artery stenosis (BCAS), RLIC can also decrease Iba1 immunostaining in the brain, suggesting that RLIC inhibits microglial activation (51). In addition, the authors reported that RLIC downregulated mRNA levels of ICAM-1 and VCAM-1, relative to those observed in the BCAS group.

##### RLIC can inhibit infiltration of peripheral immune cells into the CNS

Peripheral immune cells including neutrophils, monocytes, and lymphocytes invade the brain following acute ischemic stroke (47). Similar findings have been observed in animal models of CCCI (50, 52). CD4+ and CD8+ T lymphocytes have been observed in the brains of 2VO animals (50), and myeloperoxidase (MPO)-positive neutrophils are known to infiltrate the cerebral cortex and thalamus (52). The infiltration of peripheral immune cells aggravates inflammation in the CNS. Monocytes and macrophages can in turn release glutamate, which may then exert neurotoxic effects by activating NMDA receptors on peripheral neurons (53). In addition, CD4+ T cells secrete a variety of pro-inflammatory cytokines such as TNF-α (54).

Previous studies have indicated that RLIC attenuates the infiltration of various immune cells in the brain during acute cerebral ischemia, including monocytes (55) and neutrophils (56). Chen et al. (56) reported that MPO activity in the brain was significantly lower in the RLIC group than in the non-RLIC group. Interestingly, increased MPO activity was observed in the hindlimb gastrocnemius muscle in which RLIC was performed, indicating that RLIC may “attract” neutrophils to the muscle tissue, thereby inhibiting their infiltration into the brain (56). Other studies have reported that mononuclear cells are attracted to the limbs by CC chemokines during limb ischemia (55). Taken together, these findings indicate that RLIC may attenuate the infiltration of circulating immune cells into the brain by attracting them to the ischemic limb. However, further studies are required to determine whether RLIC exerts similar effects in CCCI.

##### RLIC can regulate immune status both in the CNS and the peripheral blood

T helper (Th) cells are T lymphocytes which are characterized by the presence of CD4 molecules on the cell surface, and can be divided into multiple subtypes including Th1 cells, Th2 cells, regulatory T (Treg) cells, and others. The overall immune response is regulated by the balance between Th1 and Th2 immune responses. Th1 cells mainly secrete interferon-γ (IFN-γ) and IL-2, leading to the aggravation of inflammation; while Th2 cells secrete IL-4, IL-5, IL-10, and IL-13, which play an important role in attenuating the pro-inflammatory response of Th1 cells.

Following acute ischemic stroke, the Th1-prone immunity in C57Bl/6 mice results in a more severe functional outcome than that observed in Th2-prone FVB mice, indicating that Th1 cells contribute to increases in brain damage (57). Other studies have reported that Th1 cells exacerbate brain infarct volume and functional deficits, while Th2 cells attenuate these effects (58, 59). In the periphery, immune cells in the blood participate in the progression of atherosclerosis, which is an important cause of CCCI and stroke. Th1 cells are the most abundant type of T cell in atherosclerotic plaques (60). Th1-relevant cytokines such as IFN-γ, IL-12, and IL-18 have been reported to promote atherosclerosis by activating monocytes, inhibiting the proliferation of vascular smooth muscle cells, and upregulating the expression of matrix metalloproteinases (MMPs) (60). However, the role of Th2 cells remains controversial. High levels of Th2 cells in the blood are independently associated with reduced common carotid intima-media thickness (61). IL-5, a cytokine secreted by Th2 cells, has also been shown to inhibit lesion formation (62). However, other studies have reported a correlation between elevated IL-5 levels and the risk of coronary events (63, 64), as well as a negative correlation between plasma IL-5 concentrations and subclinical atherosclerosis (65). These findings suggest that the pathogenic effect of Th1 cells is stronger and more evident than that of Th2 cells. Therefore, shifting the immune response toward the Th1 response may exert damaging effects in the brain and cerebral arteries.

Unfortunately, during both CCCI and acute phase of stroke, the balance of the Th immune state is skewed toward Th1 response not only in the CNS but also in peripheral blood. Indeed, MCAO significantly increases the Th1/Th2 ratio in the brain (66), while 2VO increases INF-γ levels and decreases IL-4 levels in the hippocampus, suggesting an immuno-shift in the direction of Th1 (67). Vogelgesang et al. collected T cells from patients with acute stroke and stimulated them *ex vivo*, observing increases in levels of Th1-relevant proinflammatory cytokines such as IL-6, IL-1β, and TNF-α, relative to levels observed in cells from healthy controls. Notably, levels of the Th2-relevant cytokines IL-4, IL-5, IL-8, and IL-10 remained unchanged. While these findings indicate that T cells in the blood maintain full functionality and shift toward the Th1 response in patients with acute stroke (68). The above phenomenon is only observed in acute phase of mild or moderate stroke, while the lymphocytic IFN-γ production was suppressed in patients with severe stroke (69) and a significantly lower ratio of IFN-γ-/IL-4-producing T cells was observed at the late post-acute phase of stroke (70). No researchers have studied the immune status of Th cells in blood after CCCI.

RLIC may inhibit the polarization of the Th1/Th2 ratio toward Th1. Recent research has indicated that RLIC can increase levels of the Th2-relevant cytokine IL-4 in the brains of MCAO mice (71.) while inhibiting increases in the Th1-relevant cytokines IL-6 and TNF-α in the blood (9, 71). However, RLIC also inhibits increases in the anti-inflammatory mediator IL-10 (71). Further research is required to verify these findings.

#### RLIC Can regulate autophagy

Autophagy is important for the intracellular degradation of cellular materials such as damaged organelles and abnormal proteins, leading to the basal turnover of cell components and providing energy and macromolecular precursors. Moderate autophagy can eliminate damaged mitochondria in cells and reduce mitochondria-mediated damage due to ischemic diseases (72); however, abnormal increases in autophagy may promote disease progression (73–76).

During acute cerebral ischemia, autophagy helps to eliminate damaged organelles and macromolecules that have lost their function, maintains a normal CNS environment, and exerts neuroprotective effects. However, recent studies have also reported that autophagy aggravates nerve damage following acute ischemia (75). During CCCI, most current studies have suggested that long-term chronic cerebral hypoperfusion leads to excessive activation of and impairments in autophagy, exacerbating neuronal death and cognitive impairment.

Yang et al. (73) observed microglial activation, autophagy induction, exacerbation of white matter lesions, and cognitive deficits in a 2VO mouse model of chronic cerebral hypoperfusion. In their study, treatment with the autophagy inhibitor 3-methyladenine attenuated microglial autophagy, decreased the extent of white matter lesions, and improved working memory. Chen et al. further demonstrated that autophagy aggravates neuronal damage in rats with bilateral carotid stenosis (75). In addition, Zou et al. created a rat model of chronic cerebral hypoperfusion via two-step bilateral common carotid artery occlusion, in order to examine the correlation between autophagy and neuronal damage/cognitive impairment. Chronic cerebral hypoperfusion was associated with abnormal autophagy and eventual impairments in neurological function (76).

The autophagy process can be broadly divided into two phases: the formation of the autophagosome and its degradation following fusion with lysosomes. Hypoperfusion increases levels of autophagy-relevant proteins including Beclin-1, LC3-II, and P62 in the cortex. Beclin-1 is a key component in the formation of autophagosomes, while LC3-II contributes to the elongation and maturation of autophagosomes. Therefore, increases in levels of both proteins are indicative of autophagy activation and autophagosome formation. P62 can be integrated into autophagosomes and degraded by lysosomes, and increases in levels of this protein are indicative of impaired autophagosome degradation. So increases in levels of P62 indicates that the degradation process of autophagosomes is impaired. Taken together, these findings support the notion that chronic hypoperfusion leads to excessive autophagy and increases in the number of autophagosomes. However, autophagosome degradation is impaired due to defects in lysosomal maturation, resulting in incomplete autophagy. Incomplete and abnormal autophagy leads to the inability to successfully clear damaged organelles and abnormal proteins (such as amyloid-β), causing neuronal injury, intracellular amyloid-β aggregation, and cognitive decline (76). Consistent with these findings, a recent study suggested that chronic cerebral hypoperfusion triggers the activation of autophagy while inhibits autophagosome degradation (74). In this study, 2VO rats exhibited significant increases in LC3-II protein levels in the hippocampus yet decreases in levels of LAMP-2 protein, which is associated with autophagosome clearance.

**Figure 3:**
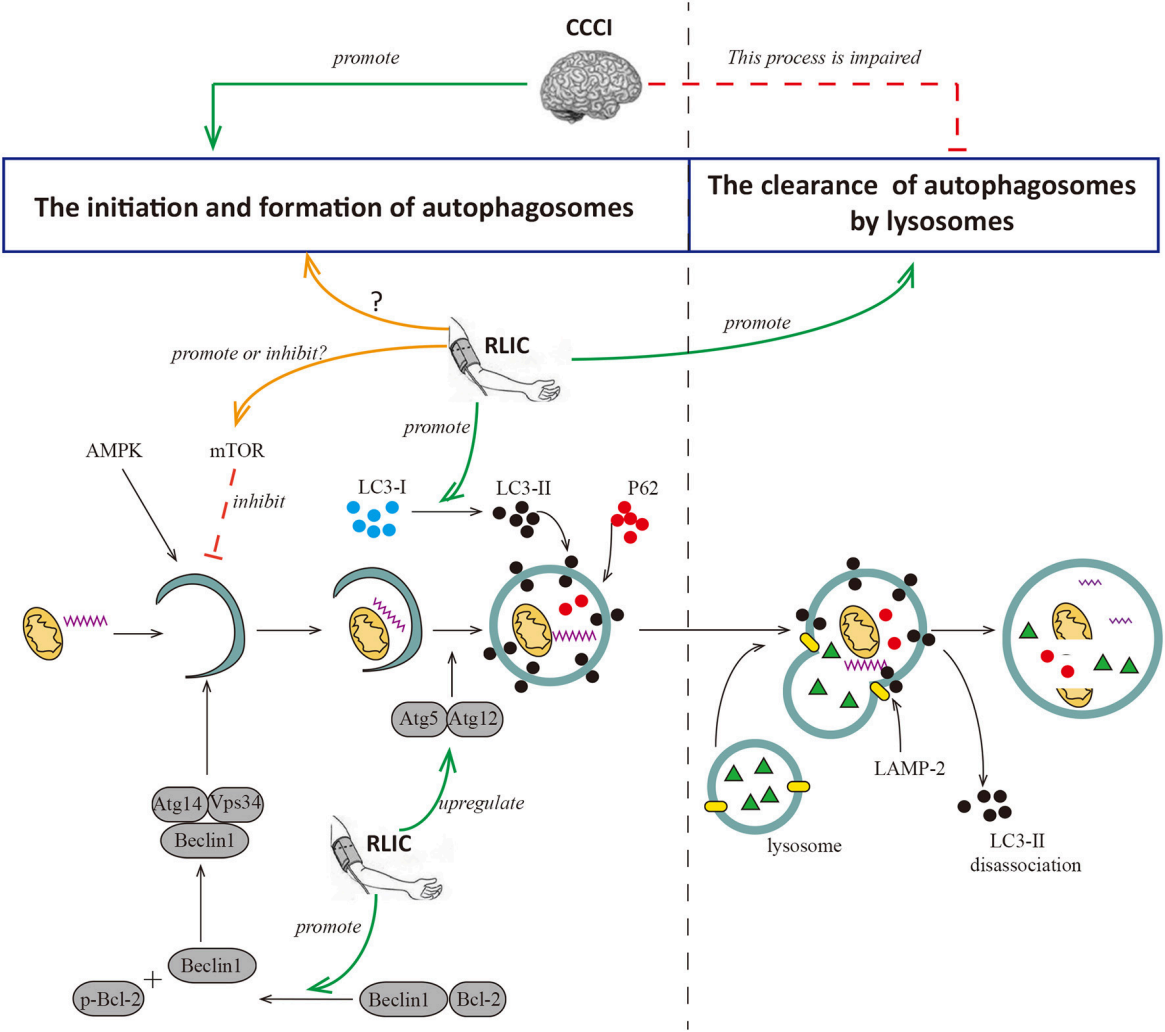
RLIC regulates the process of autophagy. Autophagy can be broadly divided into two processes, the formation of autophagosomes and the degradation of them. The former is the process of initiation, extension, and formation of autophagosomes with the participation of Beclin1, Atg5-Atg12, LC-3II, and P62, which can be inhibited by mTOR; the latter is the process of the formation of autophagy-lysosome resulted from the fusion of autophagosome and lysosome, and the degradation of it. LAMP-2 plays a key role in the formation of autophagy-lysosome; and LC-3II dissociates after the fusion of autophagy and lysosome, while P62 degrades. Cerebral hypoperfusion promotes the formation of autophagosomes, but its degradation is impaired. RLIC can regulate the activity of mTOR and affect the level of LC3-II, but different studies show different data; the levels of Atg5-Atg12 and Beclin are up-regulated by RLIC; the process of degradation of autophagosomes may be promoted by RLIC through some unknown mechanisms.

RLIC may regulate autophagy in the CNS via multiple pathways, although the results of recent studies are inconsistent. During acute cerebral ischemia/reperfusion, RLIC leads to increased levels of p-mTOR and p-p70S6K in brain tissue, and fewer autophagosomes in CNS via the activation of mTOR/p70S6K pathway, thereby contributing to decreased neurological deficits and infarct size (75). During myocardial ischemia, RLIC promotes autophagy by inhibiting the phosphorylation of mTOR, and increasing levels of the autophagosome proteins LC3-II and Atg5 (77). In addition, RLIC promotes the phosphorylation of Bcl-2 via the activation of AKT and promotes the dissociation of the Bcl-2/Beclin1 complex, thereby promoting autophagy to exert protective effects (72). Other researchers have examined the effects of RLIC on autophagy associated with CCCI. Wang et al. reported that mice subjected to BCAS exhibit increases in levels of Beclin1, LC3, Atg5-Atg12, and other proteins related to autophagosome formation in the white matter and hippocampus following RLIC (78). RLIC can also decrease levels of P62 protein, suggesting that RLIC promotes autophagosome formation, degradation, and clearance. Thus, RLIC may promote a complete process of autophagy following chronic cerebral hypoperfusion, although the mechanisms underlying this effect remain unclear (Figure 3).

### RLIC Can Improve Endothelial Function and Vasodilation in Stable Coronary Artery Disease and CCCI Caused by Large Vessel Stenosis

Endothelial cells play an important role in vascular function (79, 80). Normal and healthy vascular endothelial cells synthesize nitric oxide (NO) via nitric oxide synthase (eNOS) (79), which plays an important role in dilating blood vessels, inhibiting platelet adhesion, and promoting smooth muscle proliferation (81). Healthy endothelial cells also participate in the formation of the blood-brain barrier (BBB), blocking the infiltration of immune cells into the CNS (82). Risk factors including aging, smoking, alcoholism, and high-salt diets can lead to reduced production and bioavailability of NO, increased production of reactive oxygen species (ROS), activation and promotion of a pro-inflammatory phenotype in endothelial cells, and aggravated adhesion of immune cells such as monocytes (80, 83–85). These changes in turn result in endothelial cell dysfunction, leading to platelet adhesion, smooth muscle hyperplasia, and other adverse outcomes.

Endothelial cell dysfunction and chronic hypoperfusion constitute a vicious cycle of mutual promotion (11). Endothelial dysfunction is observed in the early stages of atherosclerosis. Atherosclerosis usually occurs in medium and large arteries, providing a common pathological basis for the development of both CCCI and stable coronary artery disease, thereby leading to chronic hypoperfusion. Furthermore, hypoperfusion promotes the differentiation of endothelial cells into a pro-inflammatory phenotype (81, 83), leads to the inflammation of nearby tissues (83) and the development of white matter lesions, accelerates the progression of atherosclerosis (81), leads to impaired microvascular vasodilation and NO signaling, and attenuates angiogenic abilities (83). One of the consequences of endothelial dysfunction is impaired vasodilation, characterized by impaired flow-mediated dilation.

RLIC has been shown to exert both microvascular and macrovascular benefits. In healthy populations, RLIC leads to improvements in skin microcirculation and brachial function based on flow-mediated dilation in both the RLIC and contralateral arms (86, 87). Notably, these effects persist even after RLIC.

Patients with chronic coronary artery disease exhibit impaired endothelial-mediated vasodilation (88). Moreover, endothelial dysfunction is an independent predictor of acute myocardial ischemia and death (88). However, long-term, regular RLIC improves the flow-mediated dilation of coronary arteries and their microvascular branches (89, 90) via an endothelium-dependent mechanism, possibly due to activation of STAT-3, increased eNOS expression, and increased CD34+ endothelial progenitor cell counts in arteries.

**Figure 4:**
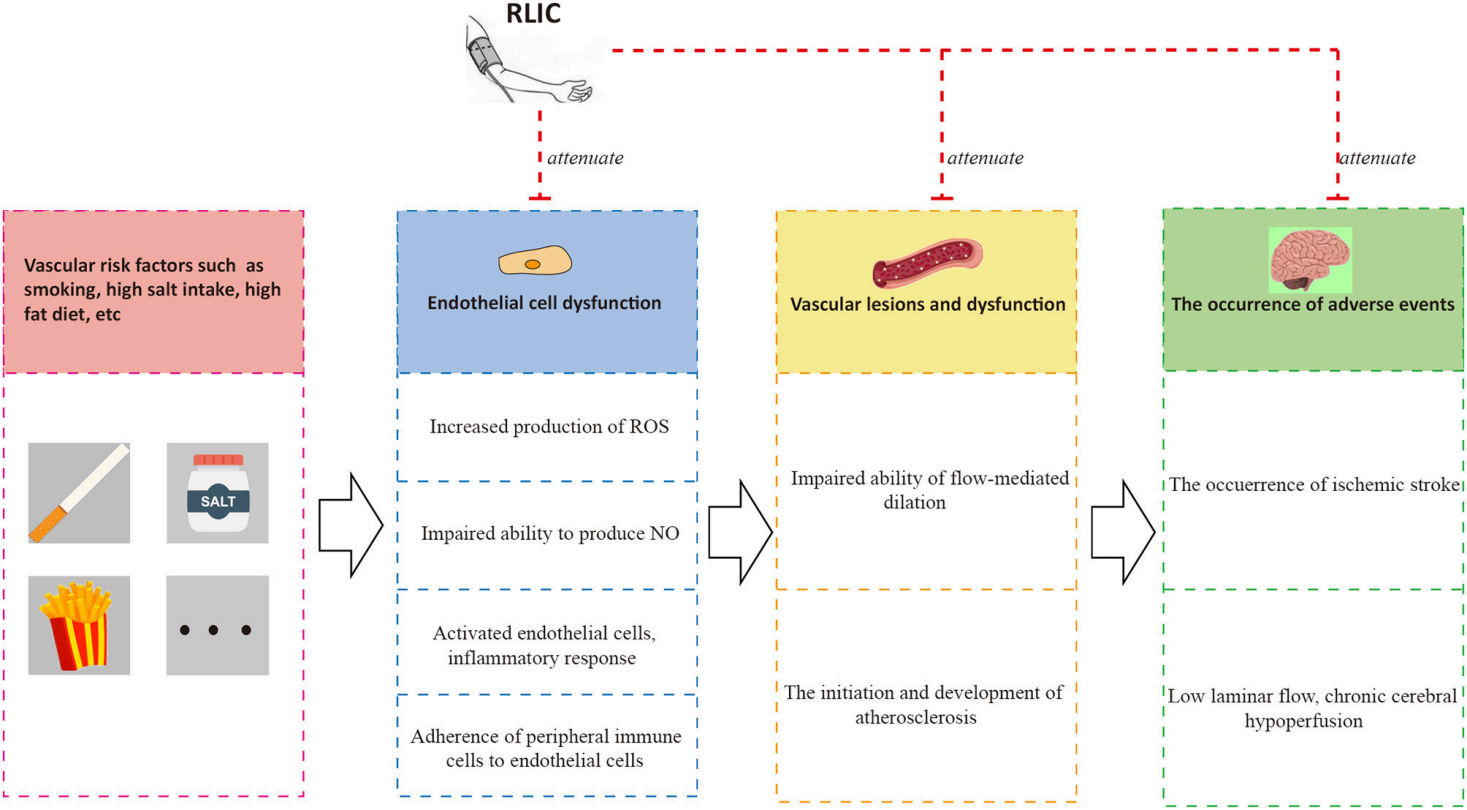
RLIC improves endothelial function. Unhealthy lifestyles such as high-salt diet and smoking can contribute to a series of pathophysiological processes of endothelial cells, leading to vascular dysfunction characterized by atherosclerosis and impaired flow-mediated dilation, which are the causes of adverse events including chronic hypoperfusion and acute ischemic stroke. The adverse events can aggravate endothelial dysfunction, forming a vicious circle. RLIC can attenuate the pathophysiological process mentioned above, and reduce the occurrence of adverse events.

RLIC may exert protective effects on endothelial function by promoting the biological activity of the eNOS-NO pathway and increasing levels of p-NOS (89, 90), reducing the production of ROS and downregulating oxidative stress (91), and attenuating inflammatory responses in endothelial cells (51). Taken together, these findings indicate that RLIC protects endothelial cells, which may in turn improve vasodilation and inhibit the progression of atherosclerosis, leading to a reduced incidence of adverse events and improved vascular circulation (Figure 4).

## RLIC May Inhibit Various Pathological Processes During CCCI Due to Other Causes

In addition to large artery stenosis, CCCI may be caused by small vessel diseases such as hypertension-induced small vessel disease, vascular amyloidosis due to Alzheimer's disease (AD), and hemodynamic factors such as hypotension and cardiac insufficiency.

### RLIC Can Alleviate Amyloid-β Deposition

AD is a neurodegenerative disease heavily influenced by genetic factors (92). The main pathological changes associated with AD include the aggregation of amyloid-β to form neurofibrillary tangles and senile plaques (11), leading to cognitive impairment and dementia. Vascular dementia (VaD) is the second most common cause of dementia after AD, mainly due to CCCI caused by vascular factors. Despite different causes, there is considerable overlap between VaD and AD with regard to disease progression (93). AD results in vascular amyloidosis (a known cause of CCCI) and aggravates cerebral hypoperfusion (16, 94). In patients with AD, decreases in CBF can be observed prior to amyloid-β deposition, suggesting that hemodynamic changes in CBF lead to impaired amyloid-β clearance and accelerated disease progression (95). CCCI due to large artery stenosis is also among the major causes of amyloid-β deposition. Indeed, BCAS mice exhibit amyloid-β deposition in the cerebral cortex (96) and small vessel walls, leading to increased thickness and fragility of blood vessel walls, narrowing of the lumen, and further aggravation of cerebral hypoperfusion (49).

RLIC can alleviate amyloid-β deposition and improve cognitive function in mouse models of CCCI due to BCAS (51). Histological experiments have further revealed that BCAS significantly increases amyloid-β levels in the frontal cortex and hippocampus, while RLIC improves cognitive function and reduces the generation and accumulation of amyloid-β (51).

Although CCCI caused by large artery stenosis induces amyloid-β deposition, the disease still differs from AD. It remains to be determined whether RLIC can inhibit the accumulation of amyloid-β and improve prognosis among patients with AD. Nonetheless, Hess et al. have argued that exercise may prevent or inhibit the progression of AD, highlighting RLIC as a “sport equivalent” (97). Thus, RLIC may be ideal for the treatment of sporadic AD.

### RLIC Can Inhibit Apoptosis of Oligodendrocytes and Reduce the Extent of White Matter Lesions

Small vessel disease has been reported to lead to chronic cerebral hypoperfusion, especially impaired CBF autoregulation and chronic white matter hypoperfusion (98). White matter lesions are also prominent in almost all subtypes of small vessel disease. In addition, hypoperfusion of the white matter due to large artery atherosclerotic stenosis can also result in white matter lesions (99), in turn leading to impaired cognitive function (13, 51). Diffuse white matter lesions are common in patients with CCCI caused by a variety of vascular factors (100). Such white matter lesions are associated with decreases in the number of oligodendrocytes, loss of myelin, and axonal abnormalities. Oligodendrocytes are widely distributed in the white matter, playing a key role in the myelination of axons and providing metabolic and trophic support to the axon. Death of oligodendrocytes leads to demyelination, resulting in slower nerve conduction and subsequent functional effects, such as motor or cognitive impairments (101, 102). Moreover, researchers have indicated that oligodendrocytes are very sensitive to ischemia and hypoxia (103).

RLIC may protect against white matter lesions following CCCI. In 2VO rats, RLIC protects against white matter lesions by activating the PTEN-Akt-mTOR signaling pathway, significantly inhibiting oligodendrocyte apoptosis, promoting myelination in the corpus callosum, and improving spatial learning 4 weeks post-2VO. While no clinical trials have examined these effects in CCCI due to large artery stenosis. But similar protective effects have been observed in patients with cerebral small vessel disease. In such patients, RLIC can alleviate white matter hyperintensities, slow cognitive decline, and significantly improve visuospatial and executive function (78). However, there are other clinical study (104) and meta-analysis (105) suggest that RLIC failed to improve the cognitive impairment. The opposite data is probably due to the small number of studies and low quality of the evidence. Thus, further clinical trials are warranted.

### RLIC Can Improve Left Ventricular Ejection Fraction

Cardiac insufficiency can lead to decreased cerebral perfusion, which in turn results in metabolic disorders and impaired cognitive function. Thus, a decrease in left ventricular ejection fraction followed by a decrease in CBF may represent the root cause of chronic cerebral hypoperfusion, which triggers a series of pathophysiological changes in the brain (14). However, previous studies have reported that RLIC improves left ventricular ejection fraction following cardiac insufficiency, prevents deterioration of the left ventricle, and improves cardiac diastolic function (106).

## Limb Ischemia Can Induce the Production of Protective Humoral Factors

**Figure 5:**
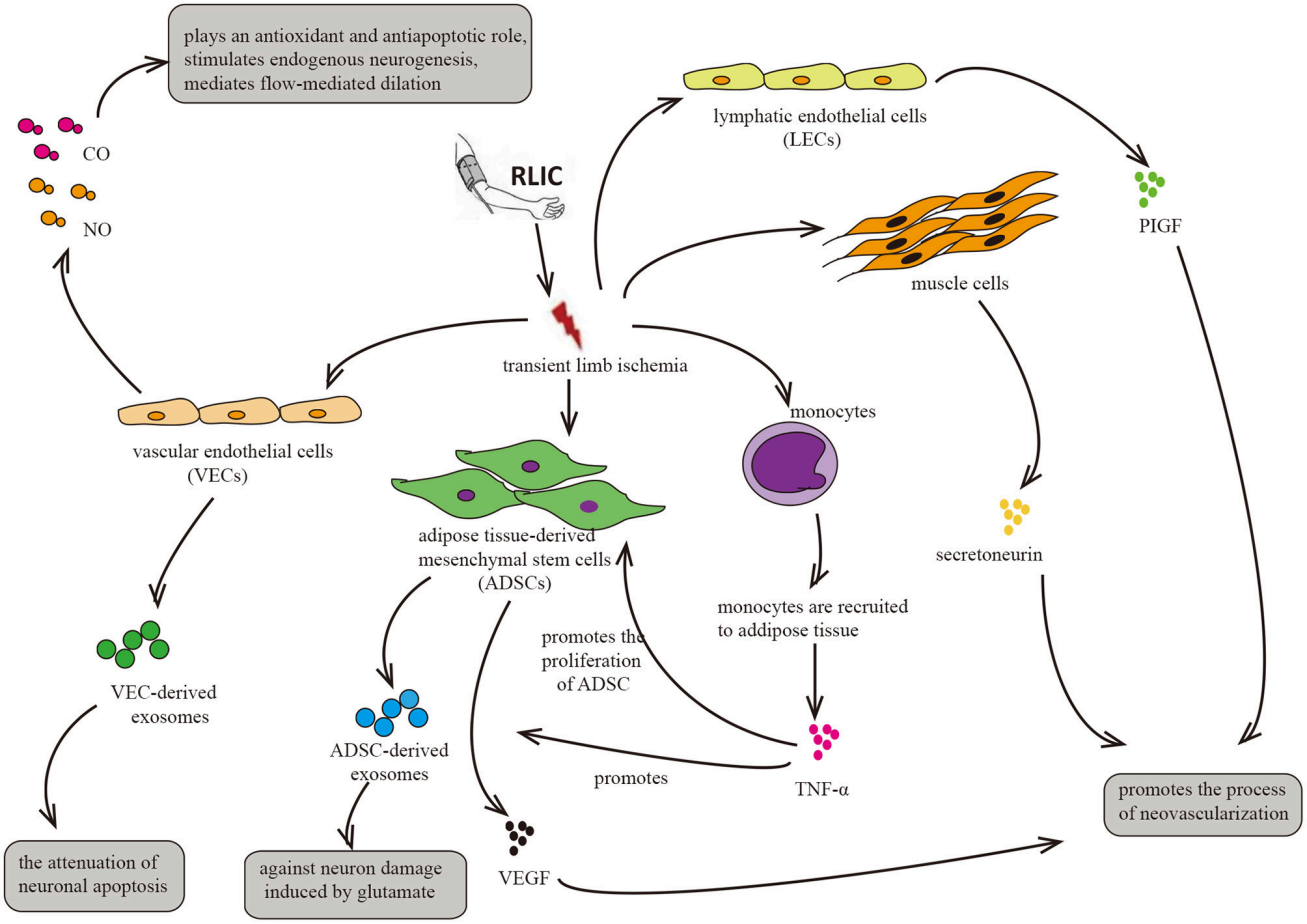
Circulatory factors that may be induced by RLIC. RLIC involves a series of transient, sublethal ischemic attacks in the limbs, which contributes to the secretion of various neuroprotective factors from different tissues and cells, including vascular endothelial cells, lymphatic endothelial cells, muscle cells, monocytes, and adipose-derived mesenchymal stem cells, eventually promoting angiogenesis and vasodilation, reducing glutamate excitotoxicity in CNS, and alleviating neuronal apoptosis.

RLIC involves a series of transient, sublethal ischemic attacks in the limbs, including muscle tissue, adipose tissue, vascular endothelial cells, and lymphatic endothelial cells. After ischemia, these tissues and cells exhibit alterations in metabolic and secretory functions, producing, and secreting humoral factors into the circulation. Such changes may underlie the neuroprotective effects of RLIC on distant brain tissue (Figure 5).

### RLIC May Induce Exosome Production

Given their important roles in the immune response and the regulation of cell signaling pathways, exosomes have recently attracted the attention of several research groups. By secreting exosomes, cells transmit signaling molecules (including miRNA and factors involved in various signaling pathways) to distant cells, tissues, or organs to exert regulatory effects.

Exosomes secreted by endothelial cells, adipose tissue-derived mesenchymal stem cells (ADMCs), and other cell types can cross the blood-brain barrier, transporting signaling molecules to the brain and exerting neuroprotective effects in various disease states (107) including acute ischemic stroke and degenerative diseases such as AD (108) and Huntington's disease (109). Thus, exosomes aid in inhibiting neuronal apoptosis, alleviating glutamate excitotoxicity, and promoting the decomposition of amyloid-β. For example, pigment epithelium-derived factor (PDEF) in exosomes secreted by ADMCs activates autophagy and inhibits neuronal apoptosis during acute cerebral ischemia (110). Exosomes secreted by endothelial cells have also been reported to exert similar effects (111). In addition, Kalani et al. observed that exosomes reduce the expression of NMDA receptors in the CNS, thereby reducing infarct volume and the degree of edema following acute ischemic stroke (112). Exosomes have also been shown to promote angiogenesis (113) as well as the degradation of amyloid-β (108), and to inhibit the loss of myelin (114, 115). While these studies highlight the protective effects of exosomes in various CNS diseases (e.g., AD (108), Huntington's disease (109), and acute ischemic stroke), no similar studies have been conducted to examine their effects in CCCI. Given the similarities in the basic pathological processes of these different CNS diseases and the seemingly universal protective effects of exosomes, we speculate that exosomes can also exert protective effects in CCCI. However, further studies are required to verify this hypothesis.

Previous studies have reported that plasma levels of exosomes increase following RLIC, noting that these exosomes may be derived from endothelial cells. Exosome production following RLIC promotes the expression of Bcl-2 and inhibits the expression of Bax at both the transcriptional and translational levels, thereby attenuating oxygen glucose deprivation/reperfusion-induced apoptosis of SH-SY5Y neurons (111). Therefore, RLIC may induce vascular endothelial cells to secrete exosomes and inhibit neuronal apoptosis in the CNS.

Exosomes are derived from various sources, including endothelial cells and ADMCs. The limbs are rich in subcutaneous fat and blood vessels, and ischemia and hypoxia in endothelial cells and adipose tissue of the limbs due to RLIC may trigger a series of relevant physiological changes. Adipose tissue plays critical roles in endocrine and immune regulation (116). However, whether RLIC can induce the secretion of exosomes from ADMCs remains to be confirmed directly. Nonetheless, previous studies have indicated that hypoxia induces the secretion of platelet-derived growth factor (117), which promotes the secretion of exosomes from ADMCs (118). Taken together, these findings support the notion that RLIC may be able to attenuate the pathophysiological changes associated with CCCI by promoting the secretion of exosomes from other cell types, although further studies are required to verify this hypothesis.

### RLIC May Induce the Production of Pro-Angiogenic Factors

Several experimental studies have demonstrated that RLIC can improve CBF in models of acute ischemia (20, 72, 119) and CCCI (120). Similar results have also been observed in clinical trials (8, 9, 25, 27).

RLIC induces ischemia and hypoxia in various tissue components of the limb, including muscle tissue, adipose tissue, vascular endothelial cells, and lymphatic endothelial cells, thereby triggering changes in metabolism and secretory functions. In addition, RLIC can induce the infiltration of immune cells from the peripheral blood into the ischemic tissues, leading to inflammation of the limbs (55, 56, 121). For example, monocytes can be recruited to ischemic adipose tissue, and ischemia promotes the secretion of cytokines such as TNF-α (55), which has been reported to enhance the proliferation of ADMCs and upregulate the expression of pro-angiogenic factors such as fibroblast growth factor 2 (FGF-2), vascular endothelial growth factor (VEGF), and monocyte chemoattractant protein 1 (MCP-1) in ADMCs (122). The secretion of these factors into the blood may exert an angiogenic effect on brain tissue. Consistent with this hypothesis, another study reported that ischemia promotes the secretion of VEGF by ADMCs (114). Similar phenomena have also been observed in clinical trials (27). In mice, hypoxia in muscle cells upregulates levels of the angiogenic cytokine secretoneurin via hypoxia-inducible factor-1alpha (HIF-1α)- dependent mechanisms (123). Other studies have reported that systemic levels of secretoneurin are significantly higher in patients with critical limb ischemia than in controls (124). Given its angiogenic effects (123–125), these findings indicate that secretoneurin may aid in the treatment of ischemic diseases. Furthermore, hypoxia activates the expression of placental growth factor (PlGF) in lymphatic endothelial cells, which is widely present in the muscles and skin of the limbs. PlGF is a member of the VEGF family, promoting angiogenesis in pathological conditions such as limb ischemia and wound healing (126).

Consistent with the aforementioned hypotheses, previous clinical studies have reported that RLIC significantly improves serum VEGF and basic fibroblast growth factor (bFGF) levels in patients with intracranial stenosis after only 10 days of treatment (27). However, significant changes in CBF were only observed after 300 days of RLIC treatment. In the long-term, RLIC may slowly improve blood flow to the distant brain by increasing serum levels of angiogenesis-related factors.

### RLIC May Induce the Production of Protective Gaseous Transmitters

Gaseous transmitters such as CO, NO, and others can be produced by vascular endothelial cells, entering the CNS via the blood circulation to exert neuroprotective effects. NO produced by endothelial cells can dilate brain blood vessels and increase CBF (127). CO plays a role in preventing oxidation and blood-brain barrier destruction (128), thereby promoting neurogenesis, anti-apoptosis, and anti-inflammation (129).

Several studies have reported that hypoxia induces the production of NO and CO from endothelial cells (130, 131). RLIC involves brief attacks of ischemia and hypoxia, which may induce the production of NO and CO in vascular endothelial cells of the limbs.

## Potential Clinical Applications of RLIC

Cognitive impairment, depression, and acute cerebral ischemia may occur following CCCI. However, research suggests that RLIC can safely improve CCCI outcomes.

### RLIC Is Safe and Well-Tolerated

Several clinical trials have evaluated the safety of RLIC. Such studies have reported that RLIC does not significantly affect blood pressure, heart rate, middle cerebral artery pulsatility index, local skin status, or plasma myoglobin levels. These findings suggest that RLIC is safe, will not affect the hemodynamics of the body or local brain tissue, and will not cause damage to local skin and muscle (8, 9, 27). As a non-invasive, safe, and well-tolerated treatment option, RLIC may aid in the treatment of CCCI in clinical settings.

### RLIC Can Improve CBF and Reduce the Incidence of Acute Ischemic Stroke

Clinical trials have reported that RLIC can improve CBF (8). However, such improvements occur slowly: Although no improvements in CBF were observed after 10 days of RLIC treatment (9), another study reported significant improvements in CBF in patients with CCCI due to intracranial artery stenosis after 300 days of RLIC treatment. Numerous studies have also noted that RLIC can inhibit the incidence of acute ischemic stroke (8, 9, 26) and significantly improve clinical outcomes, as determined based on reductions in NIHSS and modified Rankin Scale (mRS) scores (9).

### RLIC May Improve Cognitive Function

Cognitive dysfunction is among the known adverse events associated with CCCI. Several animal studies have demonstrated that RLIC can improve cognitive function in animals with chronic cerebral hypoperfusion caused by large artery stenosis (26, 120). Such improvements may be due to activation of the eNOS-NO pathway (132), decreases in amyloid-β deposition (133), inhibition of oligodendrocyte apoptosis, reduced demyelination (26, 120, 133), attenuated apoptosis due to increased Bcl-2 levels (134), autophagy regulation (78), promotion of angiogenesis, and improvements in CBF. Recent research has also indicated that RLIC can improve cognitive function in patients with cerebral small vessel disease (135). However, other studies (104) and a recent meta-analysis (105) have reported that RLIC does not improve cognitive function in patients with ischemic stroke or chronic blood vessel disease. Given the small number of studies included and the low quality of evidence (105), these conclusions remain controversial. Thus, further clinical trials regarding the effects of RLIC on cognitive function in patients with CCCI are warranted.

### RLIC May Reduce the Incidence of Depression

Dementia is usually accompanied by depression (136), with the two sharing similar neurobiological changes such as white matter lesions (137). Research has indicated that RLIC may decrease the extent of white matter lesions and the incidence of depression, although further studies are required to verify this assumption.

## Conclusion

Due to its long-term and often invisible course, CCCI has received less attention than acute cerebral ischemic stroke. However, without appropriate intervention, CCCI may lead to a variety of adverse events. Because the pathophysiological changes associated with CCCI are complex, pharmacological research in this area has been disappointing. Recent research suggests that RLIC, which is less invasive and more well-tolerated than drug treatment, can activate endogenous protective mechanisms during CCCI. In the present report, we reviewed studies related to CCCI (Table 1), as well as those related to stroke and stable coronary artery disease. The compiled evidence indicates that RLIC may exert neuroprotective effects during CCCI by attenuating the accumulation of amyloid-β in the CNS, regulating the inflammatory response, reducing glutamate excitotoxicity, attenuating apoptosis, regulating autophagy, improving endothelial cell function, and inducing the production of protective humoral factors. However, further research is required to verify whether these mechanisms contribute to the protective effects of RLIC.

**Table 1:** Studies related to CCCI.

**References**	**Animals**	**Animal models**	**Time point for RLIC**	**Major finding**
Khan et al. (51)	C57/B6 male mice	BCAS model	A week after the BCAS surgery, RILC-therapy was performed once daily for 2 weeks	RLIC improved cognitive function, inhibited inflammatory responses, prevented the cell death, reduced the generation and accumulation of Aβ, and protected WM integrity in the BCAS model
Khan et al. (120)	C57/B6 male mice	BCAS model	RILC-therapy was performed once daily after BCAS for 1 or 4 months	RLIC improved CBF, cognition, motor function, NO production, angiogenesis and arteriogenesis, and reduced white matter damage
Xu et al. (134)	Male SD rats	2VO model	After RLIC, 2VO was performed immediately	RLIC protected neurocognitive function and upregulated Bcl-2 level in the hippocampus
Wang et al. (78)	Male Wastar mice	BCAS model	RLIC was induced immediately after the BCAS for 2 weeks	RLIC upregulated mRNA level of BECN1, Atg5, and Atg7, increased activity of autophagy, and improved cognition
Li et al. (26)	Male SD rats	2VO model	3 days after 2VO, RLIC was performed once a day for 28 days	RLIC activated PTEN/Akt/mTOR signaling pathway and reduced the loss of oligodendrocytes and demyelination in corpus callosum
Ren et al. (132)	Male SD rats	2VO model	3 days after 2VO, RLIC was performed once a day for 28 days	RLIC treatment increased cerebral perfusion and p-eNOS expression in hippocampus, prevented cell death in CA1 region, and improved memory impairment

*Aß, Amyloid-β; BCAS, bilateral common carotid artery stenosis; CBF, cerebral blood flow; NO, Nitric Oxide; RLIC, remote limb ischemic conditioning; SD, Sprague-Dawley; 2VO, bilateral carotid artery occlusion*.

In addition, further studies are required to translate these experimental findings into clinical applications. Clinical studies have confirmed that RLIC can reduce the incidence of ischemic stroke in patients with symptomatic atherosclerotic stenosis (8). However, the impact of RLIC on cognitive impairment and depression in these patients remains to be reported. Nonetheless, experimental animal studies have been promising. Such studies have reported that RLIC can attenuate amyloid-β accumulation (133), reduce oligodendrocyte apoptosis, and alleviate cognitive impairments caused by large artery stenosis (26, 120). Similar protective effects have been observed in patients with cerebral small vessel disease (135), although no similar studies have been performed in patients with CCCI caused by large artery stenosis and other studies (104) and a recent meta-analysis (105) have reported that RLIC does not improve cognitive function. Depression occurs in up to 45% of patients with vascular dementia (136), and both are associated with similar neurobiological changes such as white matter lesions (137). Although RLIC can decrease the extent of white matter lesions, no clinical studies have investigated whether RLIC can reduce the incidence of depression.

Traditionally, research has focused more heavily on the protective effects of RLIC against lethal acute ischemic attacks in distant organs. However, evidence suggests that RLIC also exerts protective effects during CCCI. Although CCCI and acute ischemic stroke differ in nature, they are associated with several common pathophysiological changes, further supporting the notion that RLIC can exert neuroprotective effects in patients with CCCI. Furthermore, given the similar pathophysiological changes that occur in many diseases of the CNS, RLIC may be applicable in patients with other chronic degenerative diseases such as AD, although this research remains to be conducted. To date, only a few studies have investigated the use of RLIC during CCCI. RLIC is a low-cost, low-risk, non-invasive, and easy-to-implement method that may attenuate cognitive impairment, depression, and other adverse events in patients with CCCI. Further studies are required to verify this hypothesis, and to fully elucidate the mechanisms underlying these protective effects.

## Author Contributions

JF and DM contributed to the concept and design of this manuscript. JY wrote the manuscript and prepared the figures. LF, LB, and MX provided comments on the manuscript, modified the figures, and contibuted to the revision of this manuscript in accordance with the comments of reviewers.

### Conflict of Interest Statement

The authors declare that the research was conducted in the absence of any commercial or financial relationships that could be construed as a potential conflict of interest.
